# Deciphering the liver’s role in pancreatic cancer metastasis: pathways and therapeutic approaches

**DOI:** 10.1038/s41698-025-01202-2

**Published:** 2025-12-02

**Authors:** Jose Inzunza, Andrea C. del Valle

**Affiliations:** 1 Department of Laboratory Medicine, Karolinska Institutet, Huddinge, Sweden; 2 Department of Physiology and Pharmacology, Karolinska Institutet, Solna, Sweden; 3 Department of Microbiology, Tumor and Cell Biology, Karolinska Institutet, Solna, Sweden; 4 Department of Molecular Biosciences, The Wenner-Gren Institutet, Stockholm University, Stockholm, Sweden

**Keywords:** Cancer microenvironment, Cancer prevention, Cancer therapy, Gastrointestinal cancer, Metastasis, Cancer, Cell biology, Drug discovery, Oncology

## Abstract

The liver frequently serves as a site for metastasis in pancreatic ductal adenocarcinoma (PDAC), attributable to its extensive blood supply and supportive microenvironment, which fosters the formation of a pre-metastatic niche that facilitates tumor dissemination. Deciphering the mechanisms underlying niche formation has historically been challenging, requiring detailed investigation into the interactions between primary tumors and metastatic sites. Although these interactions were previously poorly understood, recent advances have elucidated key pathways involved in this process. These insights have been enabled by cutting-edge techniques, including spatial histological mapping, single-cell sequencing, and the identification of novel molecular markers in pancreatic and hepatic metastases. Furthermore, this review revisits and critically evaluates Paget’s ‘seed and soil’ hypothesis in light of current evidence from studies on liver metastatic niches. This review aims to deepen our understanding of liver metastasis in PDAC, with potential implications for the development of targeted therapies and improved clinical outcomes.

Pancreatic ductal adenocarcinoma (PDAC) is characterized by its high rate of liver metastases, and its five-year post-diagnosis survival rate worldwide is below 8%^1^. For patients with resected pancreatic tumors, systemic recurrence is the leading cause of death, rather than local disease^2^. This observation supports the idea that, in most cases, PDAC is a systemic disease at the time of diagnosis^3,4^. Nevertheless, not all PDACs exhibit such aggressive behavior in the early stages of development. The metastatic process is inherently inefficient. Studies in mouse models of melanoma have shown that tumors release millions of cells into the bloodstream each day, yet fewer than 0.1% of these cells succeed in forming metastases^5^. Therefore, cancer cells must overcome challenges such as exiting their primary site, entering the bloodstream, surviving stress, avoiding immune response, and adapting to unfamiliar surroundings in the host organ^6^. PDAC has a poor prognosis due to several variables, including challenges in identifying the illness in its early stages, its high risk of metastasizing, and resistance to traditional treatments. Consequently, understanding the key factors that drive pre-metastatic niche formation in the liver helps clarify how cancer cells spread and establish themselves at distant sites^7^.

The *“clonal selection model,”* originally based on Darwinian principles, suggests that tumor dissemination is driven by subpopulations of primary tumor cells that are clonally selected and expand in secondary sites^8^. However, with the advent of genome-wide transcriptome tools and their application to tumor samples, it became apparent that the clonal selection model alone was insufficient to explain the emergence of metastatic characteristics^9^. It became clear that the overall gene expression profile of primary tumors could predict the likelihood of metastasis in many types of cancer^10^. Significant intra-tumoral heterogeneity and cellular plasticity exist within tumors, enabling diverse subpopulations to acquire metastatic traits not only through clonal expansion but also by dynamic reprogramming, dedifferentiation, and reversible changes in cell states^11^. This broader and more dynamic perspective recognizes that clonal selection can occur both within the primary tumor and at metastatic sites, with polyclonal seeding and metastasis-to-metastasis spread adding further complexity. Such plasticity challenges the notion of fixed metastatic clones and underscores the importance of tumor cell adaptability and microenvironmental interactions in driving the evolution or metastasis^12^. Nevertheless, cancer metastasis is a process in which only a small percentage of malignant cells prevail rather than an evolutionary program. The mediators of metastasis play a role in this process as elements that marginally improve the likelihood of completing one or more steps of the metastatic cascade on a per-cell basis^13^. This gave rise to another well-supported theory for cancer metastasis, the “*seed and soil*,” initially mentioned by Paget and later supported by some physicians who observed that cancer cells were more likely to grow in specific organs than be widely scattered across the body. Paget’s “*seed and soil*” hypothesis, proposed in 1889, suggests that the spread of cancer cells (the *“seeds”*) to secondary sites depends on the compatibility of the target organ’s microenvironment (the *“soil”*). While this hypothesis has been foundational in understanding metastasis, recent research has revealed several challenges to its applicability across different cancer types and metastatic sites. Notably, studies in PDAC question this theory and suggest that the metastatic process may be even more complex^14^.

Liver metastasis in PDAC is particularly significant due to the unique organ-specific factors that influence tumor progression and patient outcomes. Unlike metastasis to the peritoneum and lungs, liver metastasis exhibits distinct immune landscape characteristics, including the presence of Kupffer cells, which play a crucial role in modulating immune responses and preventing metastatic growth^15^. The liver’s metabolic environment plays a crucial role in supporting the survival and proliferation of metastatic pancreatic cancer cells through metabolic reprogramming mechanisms^16^: after colonization, disseminated tumor cells in the liver adapt their metabolism to the unique hepatic microenvironment by enhancing oxidative phosphorylation and aerobic glycolysis, processes that provide the necessary energy and biosynthetic precursors to sustain rapid growth and colonization^17^. This metabolic plasticity enables PDAC tumor cells to adapt and proliferate within the fluctuating nutrient and oxygen conditions characteristic of the liver microenvironment^18^. In contrast, peritoneal PDAC metastasis is associated with severe morbidity and poor prognosis due to complications like bowel obstruction and ascites^19^, while PDAC lung metastasis often involves different immune and metabolic interactions that can impact therapeutic strategies^20^. Although many studies examine the immunohistochemical profile of PDAC’s primary tumor, systematic histopathological research on liver metastasis is uncommon. Understanding these organ-specific factors is essential for developing targeted treatments and improving patient outcomes in PDAC. Cancer metastasis to the liver involves interactions between the host immune system^21–24^, the hepatic microenvironment and cancer cells^25^.

It is now clearer that a pre-metastatic niche in the liver is established in PDAC, where hepatocytes release signaling molecules that promote cell invasion, migration, angiogenesis, and adhesion^26^. The primary question remaining is how cancer cells reprogram healthy cells to facilitate metastasis. In PDAC liver metastases, there is a notable accumulation of macrophages. These macrophages often display an immunosuppressive phenotype that promotes tumor progression and impairs anti-tumor immune responses^27–29^. Clinical studies using immune checkpoint inhibitors (ICIs), such as anti-PD-L1 and anti-CTLA-4 immunotherapy or combination therapy, have not been effective in treating pancreatic cancer^30^. In a recent phase 2 trial involving metastatic PDAC, combining ICIs (durvalumab and tremelimumab) with chemotherapy (gemcitabine and nab-paclitaxel) did not improve survival outcomes compared to chemotherapy alone^31^. The immunosuppressive tumor microenvironment (TME) in PDAC, a significant factor contributing to immunotherapy resistance, includes tumor-infiltrating immune-suppressive cells, stromal cells, and the extracellular matrix (ECM). The immune infiltration in PDAC is marked by an abundance of suppressive cells, a deficiency of anti-tumor immune cells, and immune dysfunction^32^. Hence, it is essential to comprehend the mechanisms involved in creating a pre-metastatic niche in the liver for practical efforts to prevent and treat pancreatic metastasis to the liver. This work reviews advances in the recent pathways for a pre-metastatic niche in the liver for PDAC. Moreover, it discusses the possible targeting therapies developed in the last few years. The foundation for targeting the liver metastatic niche is likely being established, providing a brief window of opportunity for effective intervention before niche formation.

## Preconditioning the niche

This review revisits and critically examines the traditional view that the liver merely provides a *“soil”* for pancreatic cancer *“seeds”* to grow as metastases. Instead, it argues that the liver actively participates in PDAC metastasis through dynamic interactions and communication with both the immune system and the primary tumor, thereby attracting and retaining circulating pancreatic cancer cells. The longstanding assumption that PDAC metastasizes early, often presenting at diagnosis, is being reevaluated in light of recent studies. Notably, mathematical modeling using exome sequencing data from matched primary and metastatic lesions suggests that the development of metastatic pancreatic cancer may take a decade^33^. This suggests that the metastatic process might not depend solely on the aggressiveness of the primary tumor. In addition, clinical cohorts have observed that the degree of liver injury is a primary protagonist in forming the encapsulating rim in metastases, its response to chemotherapy, and directly influences the patients’ long-term prognosis^34,35^. Liver metastases (regardless of their primary tumor site) are classified into three categories: desmoplastic (or encapsulation)^35^, replacement, and pushing^36–38^. The desmoplastic growth pattern of PDAC has been correlated with a better prognosis and a favorable response to chemotherapy^39^. Conversely, the replacement and pushing growth patterns are associated with a worse prognosis and show little response to chemotherapy when the tumor metastasizes to the liver^40^. Liver metastasis niches differ from other host tissues; for example, hepatic stellate cells and liver-resident fibroblasts contribute to the formation of a dense desmoplastic stroma. This stroma not only supports PDAC tumor cell colonization and growth but also impedes drug penetration, thereby increasing treatment resistance^41^. Pancreatic cancer cells exhibit dynamic interactions with hepatocytes and liver stromal cells, facilitating the exchange of metabolites and signaling molecules that actively remodel the hepatic metabolic milieu. For instance, activation of pathways such as transforming growth factor β (TGFβ) signaling by p21-activated kinase 2 (PAK2) not only drives epithelial-mesenchymal transition (EMT) but also reprograms metabolic pathways to enhance glycolysis, folate biosynthesis, and oxidative phosphorylation, while concurrently downregulating specific amino acid and lipid metabolic processes^32^. Additionally, metabolic adaptations within the liver TME distinguish it from other metastatic niches. Metastatic PDAC cells exploit liver-specific nutrients and engage in metabolic crosstalk with stromal and immune cells, aiding their survival under therapy-induced stress. The liver’s unique zonation and sinusoidal vasculature influence the extravasation of tumor cells and their spatial distribution within the metastatic niche^42^. These features suggest that the liver possesses pre-metastatic niche characteristics, which could be targeted in the development of next-generation pharmacotherapies aimed at preventing metastasis formation.

### Pro-inflammatory signaling by non-cancerous liver cells

**Fig. 1:**
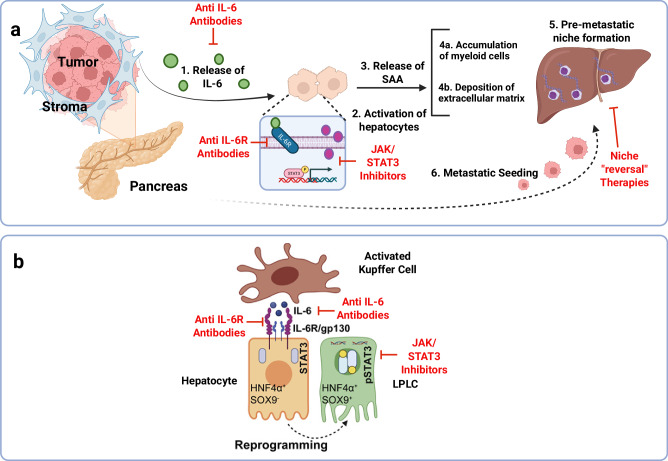
Proposed mechanism of liver niche priming by IL-6/STAT3 signaling: Kupffer cell-derived IL-6 drives hepatocyte reprogramming in response to injury or pancreatic cell influence. **a** Proposed mechanism of niche priming by IL6 and STAT3 in the liver by non-cancerous pancreatic cells. **b** Proposed mechanisms suggest that liver Kupffer cells, upon injury or inflammation, release IL-6 and promote hepatocyte reprogramming.

Lee and colleagues discovered that proinflammatory STAT3 signaling in hepatocytes is activated by the IL-6 generated by non-cancerous cells within pancreatic tumors^43^. These hepatocytes release myeloid cell chemoattractant proteins (serum amyloid A1 and A2, collectively known as SAA), which enhance fibrosis deposition by encouraging hepatic stellate cell activation. Interestingly, general inflammation brought on by IL-6 was sufficient to create a niche environment in the liver that supported the spread of cancer cells^43^. Extrapolating these results to humans, pancreatic cancer patients exhibited high circulating SAA levels associated with poor clinical outcomes^44–46^. Figure 1a illustrates this hypothesis regarding the relationship between the pancreas and liver.

Nevertheless, the generation of IL-6 is not limited to pancreatic cells alone. Recent evidence suggests that Kupffer cells in the liver respond to injury or inflammation by releasing IL-6, thereby promoting hepatocyte reprogramming^47^. Hui and coworkers recently demonstrated that, in response to injury or systemic inflammation, Kupffer cells release IL-6. They found that locally activated Kupffer cells started periportal-specific liver progenitor-like cell production. After periportal injury, mature hepatocytes reactivate genes related to reprogramming and progenitor cells, dedifferentiating into liver progenitor-like cells in both mice and humans, which significantly contributes to regeneration. Surprisingly, in vivo single-cell transcriptional profiling revealed that the pro-inflammatory factor IL-6 served as a niche signal, repurposing STAT3 activation to induce reprogramming and progenitor-related gene expression by binding to pre-accessible enhancers. Notably, reprogramming and progenitor-related genes were activated through injury-specific enhancers rather than liver embryogenesis-related enhancers. Furthermore, injury-specific enhancers, rather than those linked to liver development, were utilized to activate genes related to reprogramming and progenitor cells. These results demonstrate how inflammation-mediated transcription and a niche signal unique to damage drive the transformation of hepatocytes into a progenitor phenotype Fig. 1b.

These findings are significant for PDAC clinical care since they differ from other solid (non-blood cell) cancers due to their propensity to spread when the tumor is small^48^. This spread trait may help to explain why patients with surgically excised pancreatic tumors who do not have any visible metastases yet quickly develop liver metastases^49,50^. Surgical removal of the primary tumor can indeed lead to a temporary immunosuppressive state due to the stress response and inflammation associated with surgery^51^. This immunosuppression may reduce the body’s ability to control micro-metastatic disease, allowing these cells to grow and establish new metastatic sites. This opens the window for a new pre-metastatic niche formation therapy, such as the development of STAT3 inhibitors or antibodies that prevent IL-6 from binding to its receptor. Researchers took little time to find STAT3 inhibitors, and Yi and co-workers presented the first STAT3 inhibitor to reduce PDAC metastasis in the liver^52^. In their work, they gave a small-molecule inhibitor named N4. The mechanism of action was attributed to the N4 interactions with STAT3 in the SH2 domain, thereby inhibiting STAT3-EGFR and STAT3-NF-κB crosstalk. The suppression of the STAT3 downstream genes suppressed tumor growth and metastasis and significantly prolonged the survival of the mice.

### Pro-inflammatory signaling by pancreatic cell-derived granulocyte-MØ colonies

**Fig. 2:**
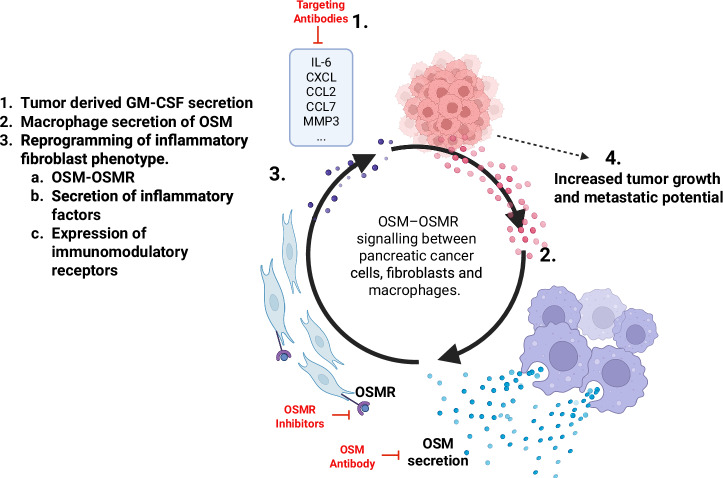
Schematic illustration of the OSM-OSMR signaling in PDAC metastasis to the liver. OSM-OSMR signaling between pancreatic cancer cells, fibroblasts, and macrophages accentuates tumor growth and metastasis.

From another perspective, additional origins of inflammatory mediators contribute to the establishment and support of the pre-metastatic niche. Some researchers have attributed the generation of inflammatory mediators, such as the IL-6 family members, to the non-cancerous cells in PDAC^43^. Others have suggested that tumor cell-derived granulocyte-MØ colonies are the result of the pro-tumorigenic inflammatory mediators by inducing expression of Oncostatin M (OSM) in MØs^53^. This was proven by injecting pancreatic cancer cells orthotopically into immune-competent mice lacking Osm (Osm−/−). Results showed that knocking down the gene decreased tumor size and exhibited an altered microenvironment. Moreover, the increased abundance of αSMA-positive myofibroblasts was accompanied by a reduction in IL-6, CXCL1, TNF-α, and GM-CSF. The cognate OSM receptor later explained that OSMR is expressed in mesenchymal stromal cells, mainly fibroblasts and perivascular cells (Fig. 2)^54^. Researchers noted that the OSMR expression level is correlated with overall inflammatory gene expression, immune infiltration in the tumor microenvironment, and poor overall survival in humans with PDAC. Although no chemotherapeutic drug currently targets specific granulocyte-MØ cells, this opens the door for further research on gene delivery therapeutics and the development of antibodies targeting pro-inflammatory cytokines to reduce pancreatic metastasis to the liver.

### PDAC-derived exosomes

**Fig. 3:**
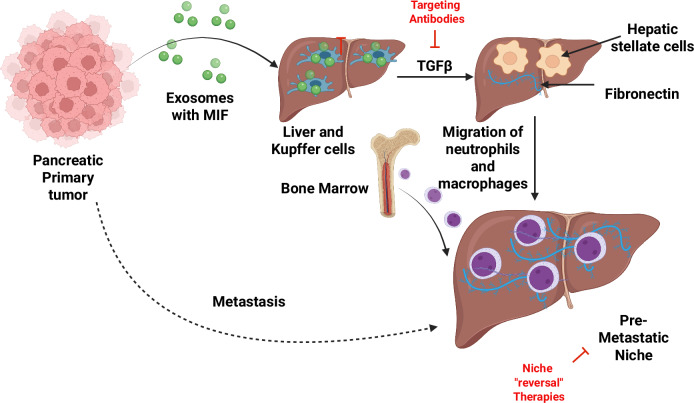
Primary pancreatic tumor cells release exosomes containing MIF into the bloodstream. These exosomes are derived from PDAC and are selectively taken up by liver Kupffer cells. This uptake leads to the production of fibrotic cytokines by the Kupffer cells, which are dependent on MIF. These fibrotic cytokines, particularly TGFβ, activate liver hepatic stellate cells to produce fibronectin. The deposition of fibronectin in the liver leads to the formation of a fibrotic microenvironment that promotes the recruitment of bone marrow-derived cells such as macrophages and neutrophils. These sequential events establish a pre-metastatic niche, which allows the survival and proliferation of disseminated PDAC cells and the formation of metastases in the liver.

The recent discovery of PDAC-derived exosomes that induce a liver pre-metastatic niche has been a recent discussion in the treatment of PDAC^55–57^. Bruno Costa-Silva and co-workers conducted a preclinical investigation in naïve mice where PDAC-derived exosomes increased metastatic liver burden. Results showed that the exosomes were primarily taken up by Kupffer cells, leading to the secretion of TGFβ and the upregulation of fibronectin in hepatic stellate cells. The fibronectin deposits promote the recruitment of bone marrow-derived macrophages and neutrophils in the liver. Careful observation of the exosomes showed that they also contained a macrophage migration inhibitory factor (MIF), and its knockdown prevented liver pre-metastatic niche formation (Fig. 3). These results were further validated by comparison with clinical samples from patients whose pancreatic tumors did not initially progress; notably, MIF concentrations in PDAC-derived exosomes were elevated in those patients who subsequently developed liver metastases. These exciting results suggest that the PDAC-derived exosomes prime the liver for metastasis. They could serve as biomarkers and as functional components for developing therapeutics targeting pre-metastatic niche formation.

### External factors aiding niche formation

In addition to the pathways involved in priming the liver to host cancer metastasis, it is crucial to recognize the factors that influence the colonization of PDAC in the liver. Early cancer studies primarily focused on the primary tumor and its immediate microenvironment^58–60^, but they overlooked the complex interactions among PDAC cells, the immune system, and liver cells. This narrow focus constrains the understanding of the interactions between PDAC cells and the immune microenvironment, particularly within the unique hepatic cellular landscape. Recent studies have demonstrated that these interactions are pivotal for metastasis. For example, primary tumors release circulating tumor cells (CTCs), which then settle in distant metastases. To understand the mechanism by which CTCs evade immune monitoring, researchers have studied the transcriptomes of primary liver metastases and CTCs from human PDAC. It was discovered that the immunological checkpoint between Class I histocompatibility antigen, alpha chain E (HLA-E) and CD94-NKG2A was a major protagonist in the interaction between the CTC and natural killer cells. In vitro and in vivo results demonstrated that this connection is disrupted when NKG2A is blocked or when HLA-E expression is reduced. This improved natural killer cell-mediated tumor death in vitro and inhibited tumor spread in vivo. These results were attributed to the AKT-GSK3-CREB signaling pathway, where the RGS18 derived from platelets enhanced HLA-E expression, which subsequently aided in pancreatic tumor liver metastases^61^.

Additionally, PDAC tumors significantly remodel the liver TME by orchestrating a complex network of cellular interactions and molecular pathways. Central to these changes are cancer-associated fibroblasts (CAFs), regulatory T cells (Tregs), tumor-associated macrophages (TAMs), and myeloid-derived suppressor cells (MDSCs), which collectively promote a tumor-supportive environment. In addition to the IL-6/STAT3 signaling axis, discussed previously, other critical mechanisms driving this reprogramming have been found recently, including the TGFβ signaling^62^, chemokine pathways involving CXCL/CXCR^63^, and ECM remodeling mediated by matrix metalloproteinases (MMPs) and integrin β1. These interactions enhance immunosuppression, facilitate ECM deposition, and support the formation of metastatic niches. Moreover, TAMs and metastasis-associated macrophages secrete granulin, which activates hepatic stellate cells and induces hepatic fibrosis, while CAFs further modulate local immune responses and contribute to drug resistance.

This dynamic is further influenced by metabolic crosstalk and the secretion of factors such as osteopontin, periostin, and SAA, which collectively adapt the liver stroma for effective tumor colonization^64^. The PDAC-specific genomic landscape and significant epigenetic changes, including mutations in key genes such as TP53, MYC, and CDKN2A, as well as epigenetic modifications like DNA methylation and histone acetylation or repression, crucially alter the secretory profiles and gene expression in PDAC cells^65^. These alterations subsequently shape the hepatic microenvironment, thereby enhancing metastatic potential.

For instance, the loss of epigenetic regulators, including KDM6A and p300, alongside the activation of pioneer transcription factors such as FOXA1 and p63, promotes invasive behavior and facilitates metabolic adaptation at metastatic sites^66^. Moreover, pre-existing liver conditions, such as cirrhosis, hepatitis, and non-alcoholic steatohepatitis (NASH), emerge as independent risk factors for PDAC liver metastasis, likely due to the establishment of an already inflamed or fibrotic liver, which is more conducive to supporting pre-metastatic niches^67^.

Notably, the liver’s metabolic milieu and its immune regulatory functions substantially impact the behavior of metastatic PDAC cells. Understanding these interactions is crucial for designing more effective therapeutic strategies that target both the primary tumor and metastatic sites, as well as their unique microenvironments. Clinical evidence later indicated that these interactions contribute to the aggressive phenotype of cancer and increase resistance to conventional therapies^68^. Therefore, the interactions between cancer cells, the immune system, and host liver cells play a significant role in the successful metastasis of PDAC to the liver. Understanding the major pathways involved in PDAC-liver metastases is essential. This knowledge could lead to the development of new therapeutic strategies targeting the tumor microenvironment and controlling the formation of liver metastasis.

### Cancer-associated fibroblasts, collagen, and growth factors: the stroma

One of the significant challenges compromising therapeutic outcomes in PDAC is desmoplasia^69,70^. This desmoplasia is often seen in biopsies of PDAC tumors and metastases. CAFs are one of several cell types of the desmoplastic stroma, the primary source of ECM in the TME^71^. According to emerging evidence, the thick collagen matrix is thought to provide resistance to anti-PDAC treatments^72,73^. Additionally, activated CAFs release paracrine ligands that stimulate angiogenesis and immunosuppression, thereby facilitating the development of tumors. As a result, numerous pharmacological targets have been discovered for reducing CAF activation, such as losartan, an angiotensin II receptor blocker that lowers tumor fibrosis by reducing TGFβ^74^. When the TGFβ inhibitor galunisertib is combined with gemcitabine, it increases survival in patients with unresectable pancreatic cancer. Galunisertib and duravalumab were combined in another phase I trial, and this time, the tolerability and safety profile were satisfactory^75^. Hence, a further understanding of how CAFs interact with the primary tumor and their metastasis can promise a better treatment approach for PDAC and reduce pancreatic metastasis to the liver.

To understand how CAFs influence therapeutic resistance, a recent study demonstrates that chemotherapy increases placental growth factor (PlGF), directly stimulating CAFs to cause fibrosis-associated collagen deposition in PDAC^76^. In this study, patients with a poor prognosis exhibited higher PIGF/VEGF expression, more CAFs that expressed the PIGF/VEGF receptor, and greater collagen deposition. Single-cell RNA sequencing revealed that the CAF population decreased after treatment with an engineered multi-paratopic VEGF decoy receptor (Ate-Grab) and gemcitabine. These findings suggest a combined therapeutic approach for desmoplastic disease and elucidate the mechanism underlying chemotherapy-induced fibrosis in PDAC. However, the mechanism by which inflammation was caused in CAFs remained unknown until Lee and coworkers demonstrated that fibrosis in PDAC is driven by hypoxia^77^. In this case, IL-1 expression was necessary for the development of inflammatory CAFs (iCAFs) that were strongly induced by hypoxia in the tumor. Researchers also found that iCAFs preferentially reside in hypoxic areas and contribute to the heterogeneity of PDAC. Nonetheless, the direct relationship between tumor hypoxia and the pre-metastatic niche in the liver remains unknown. Cancer cells may modify the liver’s pre-metastatic niche directly or indirectly by changing the levels of oxygen and glucose in the liver^78–81^. This leads to anaerobic metabolism and lactate production, which can promote tumor growth and progression, more commonly known as the *Warburg effect*^77^.

Nevertheless, the above studies on CAFs primarily focused on the pancreatic tumor microenvironment rather than their metastasis to the liver. The relevance of similar metastasis-associated fibroblasts (MAFs) has been evidenced only in vivo studies using untargeted treatment with the anti-angiogenic medication sunitinib. This study demonstrated that MAFs play a role in mouse hepatic metastases. Despite being smaller and containing fewer stromal cells, treated metastases could still sustain liver angiogenesis and metastatic production^82^. Additionally, sunitinib failed to reduce MAFs and other stromal cells. Researchers used in vitro co-culture systems to investigate the interactions between metastatic pancreatic cancer cells and fibroblasts. Co-cultures stimulated angiogenesis and increased the proliferative rate of fibroblasts. The cause of angiogenesis was attributed to the release of IL-8 and IL-2 by MAFs. Overall, MAFs may be promising therapeutic targets, underscoring the importance of further research on the relationships between stroma and tumor development, as well as their metastasis.

## Perspectives

Forming a pre-metastatic niche in the liver is a complex process involving the interaction between cancer cells, hepatocytes, and other cell types. Previous theories of metastasis in PDAC were tumor-centric and omitted the interactions with different systems across the body, focusing solely on the complexity of pancreatic cancer tumor biology. Although interesting, the sole classification of PDAC into tumor (classical/basal) and stroma (inactive/active) subtypes has not led to an efficient therapeutic approach^83^. Recent advancements have shifted this perspective towards a more comprehensive understanding of metastatic disease research that has emerged in recent years, highlighting the critical role of the liver in facilitating tumor invasion^84^. Although the current studies have been focused on the pathways driving metastasis and invasion, they still rely on a more tumor-centric perspective, with little known about the role of hepatocytes in tumor invasion and the growth patterns in metastasis. However, the factors that drive different growth patterns and how these can be targeted via pharmaceutical means remain unknown. Here, we discussed the mechanisms explored for the formation of the pre-metastatic niche in the liver, providing an insight into the potential targeting pathways in the liver’s role in promoting PDAC metastasis. Nevertheless, further research is essential to elucidate the mechanisms by which hepatocytes orchestrate the formation of pre-metastatic niches in the liver. Advancing our understanding in this area is critical for the development of targeted therapeutic strategies to inhibit niche formation, progression, and dissemination of metastases^85^. PDAC tumors frequently exhibit low tumor mutational burden and limited neoantigen expression, which further reduces immune recognition and diminishes responses to checkpoint inhibitor therapies. Additionally, tumor cells in PDAC downregulate major histocompatibility complex class I molecules, impeding antigen presentation and immune surveillance^86^. The stroma not only forms a physical barrier but also produces immunosuppressive cytokines (e.g., TGFβ, IL-10) and metabolites (e.g., adenosine, lactic acid) that further suppress T cell function and promote immune tolerance within the TME. Clinical trials have demonstrated disappointing response rates with both single-agent and combination checkpoint inhibitors in PDAC, except in rare cases of mismatch repair deficiency or high microsatellite instability (MSI-H)^87^. Most patients with metastatic PDAC, especially those with liver involvement, experience minimal benefit because metastatic niches have additional unique immunosuppressive cues. To date, few clinical studies have been conducted that expand PDAC treatment beyond the tumor-centric approach, and interesting results have been observed with IL-6/STAT3, TGFβ, and CSF1R inhibitors, as displayed in Table 1. Current research is now focused on strategies to overcome stromal barriers and to “reprogram” or deplete immunosuppressive cell populations within the TME, such as using CD40 agonists, CSF-1R inhibitors, or therapies targeting cancer-associated fibroblasts. Ultimately, combination therapies that address both tumor-intrinsic resistance and extrinsic microenvironmental suppression are considered the most promising avenues for enhancing immunotherapy efficacy in PDAC. Nevertheless, hepatic transcriptional profiling of PDAC liver metastases reveals substantial inter-patient heterogeneity, encompassing differences in immune cell composition, stromal activation, and metabolic pathways. Moreover, there are inherent risks and limitations: perturbing the liver microenvironment could disrupt normal hepatic functions, potentially causing toxicity or impaired regeneration. Additionally, the complexity and plasticity of TME cells pose challenges for achieving durable reprogramming. These considerations underscore the importance of developing carefully designed therapeutic strategies with precise targeting to maximize benefits while minimizing adverse effects.

**Table. 1:** Current clinical trials related to therapeutic targets in PDAC

Therapeutic target	Clinical trial	Phase	Drugs used	Current status
IL-6/STAT3 Inhibitors	Phase I Trial of TTI-101, a first-in-class oral inhibitor of STAT3 in patients with advanced solid tumors	Phase I NCT03195699	TTI-101	Ongoing, recommended phase II dose established
IL-6/STAT3 Inhibitors	Phase II Trial of Danvatirsen (AZD9150) in combination with Durvalumab for advanced solid tumors	Phase II NCT02983578	Danvatirsen, Durvalumab	Ongoing, evaluating efficacy and safety
TGFβ inhibitors	Phase II trial in combination with chemotherapy, advanced or metastatic unresectable pancreatic cancer	Phase II NCT01373164	Galunisertib (LY2157299)	Completed, phase II dose established
CSF1R inhibitors	Phase I trial, patients with metastatic/advanced pancreatic or colorectal cancer	Phase I NCT02777710	Anti-PDL1 antibody (Durvalumab) combined with CSF-1R TKI (Pexidartinib)	Ongoing, evaluating efficacy and safety

Finally, the patient’s diet and metabolic activity could also impact the priming of the liver’s pre-metastatic niche. A recent study on high-fat diets has shown that they can prime organs to receive cancer cells and facilitate the growth of secondary tumors. Researchers found that mice with breast cancer fed a high-fat diet had more lung metastases and exhibited more ‘activated’ platelets in their lungs than mice fed a regular diet. However, this study has been conducted using murine breast cancer models^88^. It would be interesting to understand how the liver-PDAC interaction is influenced by other factors such as diet, circadian rhythm, and physical activity. Investigating these interactions can reveal novel therapeutic targets and enhance personalized treatment strategies for patients with PDAC. Finally, some studies have demonstrated that the microbiome can influence metastatic behavior by affecting inflammation and immune cell activity. Specific microbial communities in the gut and tumor tissue have been linked to alterations in metastatic potential and patient prognosis^62^. Within PDAC tissue, the microbiome can modify the tumor microenvironment, creating conditions that favor cancer cell survival and proliferation^89^. For example, bacterial populations have been found to modulate immune responses, potentially aiding cancer cells in evading the immune system^90^. Understanding the microbiome’s role in PDAC can lead to novel therapeutic strategies. Nevertheless, careful examination of research procedures is crucial when studying the tumor microbiome, since several published microbiome studies have been recently questioned regarding protocol integrity and sample contamination^91,92^.

## Conclusion

This review critically examines the mechanisms underlying PDAC metastasis, with an emphasis on pre-metastatic niche formation in the liver. The hepatic microenvironment, characterized by its extensive blood supply, facilitates the colonization and proliferation of metastatic cancer cells. Recent advancements using spatial histology mapping, single-cell sequencing, and novel molecular marker identification have elucidated key pathways involved in niche establishment and tumor-host interactions. These studies have provided profound insights into the liver-tumor interface, highlighting complex interactions among cancer cells, immune components, and resident hepatic cells. Despite these advances, several crucial questions remain unresolved. Notably, the specific roles of immune cell subsets, such as Kupffer cells and T lymphocytes, in modulating the metastatic niche warrant further investigation. Additionally, the influence of hepatic metabolic reprogramming on cancer cell viability and growth is also not fully understood. Future research should focus on delineating the functions and mechanisms of immune cells at the liver-tumor interface to clarify their contributions to niche formation and metastatic progression. Additionally, exploring the metabolic vulnerabilities of PDAC cells within the hepatic milieu could reveal therapeutic targets to impede metastasis. Development of advanced preclinical models that emulate the heterogeneity and complexity of human PDAC metastasis is essential to translate findings effectively. Combining therapies that target pathways involved in niche establishment, including immunomodulation, metabolic reprogramming, and microbiome interactions, may improve clinical outcomes. The review underscores the emerging significance of adjuvant and neoadjuvant therapies in PDAC management and advocates for integrated strategies to improve patient prognosis. Addressing these unresolved questions and pursuing the directions outlined will advance our understanding of PDAC metastasis and promote the development of more effective therapeutic interventions.

## Data Availability

No datasets were generated or analysed during the current study.
